# Quality of Life in Patients with Thalassemia Major and Intermedia in Kerman-Iran (I.R.)

**DOI:** 10.4084/MJHID.2012.058

**Published:** 2012-10-03

**Authors:** Hossein Safizadeh, Zahra Farahmandinia, Simin Soltani nejad, Nasim Pourdamghan, Majid Araste

**Affiliations:** 1 MD. MPH, Neurosciences Research Center, Kerman University of Medical Sciences, Kerman, Iran; 2 MD, Department of pediatrics, Afzalipour Medicine School, Kerman University of Medical Sciences, Kerman, Iran; 3 Medical Student Research Committee, Kerman University of Medical Sciences, Kerman, Iran; 4 MD, Research Center for Social Determinant of Health, Kerman University of Medical Sciences, Kerman, Iran; 5 MD, Iranian Thalassemia Society, Kerman, Iran

## Abstract

Thalassemia is the most common hemoglobin disorder in the world and thalassemia major and intermedia stand among the most severe forms. Due to recent improvements in treatment, patients with thalassemia have longer life expectancies; hence it is of utmost importance to pay careful attention to their quality of life together with life expectancy. This study was conducted to assess the quality of life in patients with thalassemia and also to compare it between thalassemia major and intermedia. In this cross-sectional study, patients who referred for blood transfusion or follow-up visits were evaluated for their quality of life (QOL). Short Form-36 questionnaire was applied to evaluate QOL. In this study, 308 patients with a mean age of 22.95±4.82 years were evaluated. The scores of QOL were regarded as moderate in eight domains under evaluation; the least score was given to General Health (53.05±16.96) whereas the highest score was given to Physical Functioning (67.95±22.68). The QOL in the patients with thalassemia major was better than those with thalassemia intermedia regarding Physical Functioning and Role Limitation Emotional domains. Compared to injecting chelators, patients who received oral chelators showed to have a better QOL considering Social Functioning and Mental Health domain. The patients under study didn’t have a satisfying QOL ; the QOL of patients with thalassemia major was better than that of patients with thalassemia intermedia in only 2 domains of sf-36(Physical Functioning & Role limitation-Emotional). It is then essential that experts pay proper attention to improve QOL among patients.

## Introduction

Thalassemia as the most common genetic disorder worldwide, is regarded as a serious problem in public health issues in the Mediterranean region.^1^ Iran is located in the geographical belt of thalassemia and it has been estimated that thalassemia carriers vary from one to ten percent (with a mean of 4.5%) in different parts of Iran. According to the statistics, the number of patients with thalassemia major (the most severe form of thalassemia) in Iran is more than 20,000 individuals.^2^ Thalassemia affects the synthesis of globin chain and according to the number and type of the involved chains, clinical symptoms vary. Beta-thalassemia is the most common frequent type of thalassemia which presents in three forms: thalassemia minor which leads to a mild asymptomatic hemolytic anemia, thalassemia intermedia and major are more sever forms. Interestingly, thalassemia major is the most severe form of beta-thalassemia leading to severe anemia and patients are in need of blood transfusion since the young age; this type may end in heart failure, due to iron overload, or early death in childhood in case of no transfusion. Thalassemia intermedia appears later in life with milder symptoms and its presentations include hepatomegaly and moderate to severe anemia; this latter form needs less consecutive blood transfusion.^3^

Nowadays, with improvements in the treatment of patients with thalassemia, these patients have a longer life expectancies and a larger number of them reach older ages.^4^ As a result of this increase in life expectancy, their needs also change and entities such as continuing education, career and family making become more prominent amongst them.^5^ It is then anticipated that effective and suitable life background would be prepared for them in the society and they become as active as other members of the society.^6^ Such an increase in life expectancy is accompanied by certain challenges such as bone diseases, infertility, consecutive referrals for blood transfusion, subcutaneous infusion of chelators, and moral stresses^5^ in a way that these aforementioned problems would affect mental, physical, social and educational functions of these patients.^1^ Hence, authorities who provide services to patients should be aware of the related mental and social consequences in addition to the burden of this disease, just like any other chronic disease, to prepare better living environment for these patients.^6^ Therefore, this study aimed to evaluate the quality of life amongst patients and to compare it between thalassemia major and intermedia.

## Material and Methods

The proposal of this study has been approved in the research committee of Afzalipour School of Medicine and the Ethics Committee. In this cross-sectional study, samples included patients with thalassemia major and intermedia in Kerman city (the center of the largest province in Iran). Kerman is one of the populated cities in Iran and socioeconomically it is comparable to other major cities of Iran such as Shiraz. Three hundred eight patients, referred to the special treatment center for thalassemia for blood transfusion or treatment follow-ups, entered the study, sequentially. Routinely, in this center patients with thalassemia major refer according to a predefined schedule (at least once monthly) but patients with thalassemia intermedia receive blood transfusion as prn. Either subcutaneous Desferroxamine or oral Deferasirox was prescribed for each patient as chelation therapy. They aged sixteen years or older. Informed verbal consent was requested and the confidentiality of information was ensured. Short Form-36 (SF-36), a general questionnaire for assessing health related quality of life, was applied to assess the patients’ QOL. Such a questionnaire assesses eight domains of QOL including physical functioning (PF), role limitation physical (RLP), bodily pain (BP), general health (GH), vitality (V), social functioning (SF), role limitation emotional (RLE) and mental health (MH). The score given to QOL in each domain varies between zero to 100; a score close to zero implies a worse QOL and the one close to 100 shows a better QOL.^7^ The Persian version of this questionnaire is available and its reliability and validity have already been confirmed in order to be applied in assessing QOL amongst patients with thalassemia.^8^,^9^ Information about complications was collected based on self-report.

Differences were tested for significance using t-test for the comparison of continuous variables between 2 groups, ANOVA for the comparison among multiple groups and Tukey’s test for pairs of groups. Correlation among continuous data was performed using Pearson correlation and relations between variables were assessed using linear regression. A significant level was defined when P<0.05. Data were analyzed by SPSS17.

## Results

Three hundred eight patients were evaluated in this study. Their demographic characteristics are presented in table 1. Two hundred nine (67.9%) and 99 individuals (32.1%) had beta thalassemia major and intermedia, respectively. Most of the patients (85.4%) used subcutaneous desferroxamine and only a minor proportion (4.9%) presented complications (Table 1). The reported complications were Diabetes Mellitus (n=9), musculoskeletal complications (n=3), Hepatitis C (n=1) and GI upset (n=1). The patients with thalassemia major were similar to those with thalassemia intermedia for age; hemoglobin level of the last four measurements was higher in the patients with thalassemia major than in those with thalassemia intermedia (9.7mg/dl versus 8.9mg/dl) (p<0.05). Moreover, there was a significant difference in the serum ferritin level in the last two measurements (3248.64 in thalassemia major versus 2400.88 in thalassemia intermedia) (P<0.05).

**Figure 1:**
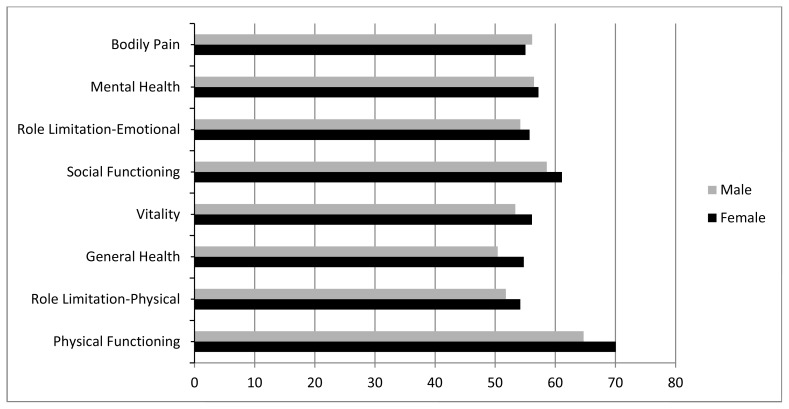
SF-36 Domain scores in male and female patients.

The scores of QOL in all eight domains were moderate presenting PF with the highest score (with a mean score of 67.95) and GH and RLP with the lowest scores. According to the analysis, although the female participants had a higher score in all domains except for BP, when compared to the male participants, such a difference was only significant in PF and GH domains (p<0.05)(Figure 1). There was significant difference regarding income per month only in RLP domain and patients with 5 million Rials and above had better QOL (P<0.05), but there was no significant difference regarding different age groups and educational levels in any of the domains.

Comparison between the QOL in the patients with thalassemia major and intermedia revealed a difference in three domains (Table 2) in a way that PF and RLE were worse in those with thalassemia intermedia whereas BP was better amongst them(p<0.05).

Comparing the patients according to the received chelator also showed that the score of those with oral chelators was higher than those with injecting chelators in all domains except for PF(Table 3); such a significant difference in quality was observed in GH, V, SF, BP (p<0.05) and MH (P=0.05). The domains were not significantly different in the patients with and without complications. The results of Pearson’s test showed no correlation between the QOL domains and the mean level of hemoglobin and ferritin as measured in the recent laboratory tests.

To eliminate the effect of confounding factors, multivariate linear regression was applied (Table 4); according to this analysis, the female patients’ QOL was better only in GH domain when compared to the males and the QOL in the patients with thalassemia major was better solely in PH and RLE in comparison with thalassemia intermedia. The QOL of the patients who received oral chelators was better in SF and MH compared to injecting chelators’ receivers. Regarding the effect of income on the QOL, it was revealed that those with an income more than five million Rials a months had a better QOL compared to those with a less amount of income, in RLP domain; in addition, regarding SF domain, the participants with a high school diploma or a higher educational degree had a better QOL in comparison with those who had a lower educational degree.

## Discussion

The QOL amongst the patients with thalassemia in Kerman is moderate in eight domains under study; yet, it is not regarded as very good compared to the rest of Iranian healthy members (Table 1).^8^ Such a difference is not surprising since due to special physical conditions and problems related to their disease, patients are not anticipated to have a very good quality of life.^6^ The results of a study conducted by AzarKeivan on the QOL amongst patients affected with thalassemia (major and intermedia) aged eighteen years and older, in Tehran city revealed that they had a better QOL in all domains except for MH(56.7±17.4) and BP(56.4±17.0) compared to this present study;^10^ in her study the highest score was seen in PF and SF, and the lowest score in BP and MH. Another study conducted by Hadi et al. in Shiraz has demonstrated a better QOL in all domains compared to this study, the highest score was seen in RLP and Pf and the lowest score in GH and V.^11^ The only common point between these two studies and the present study was in PF domain which was better than the other domains; hence it seems that receiving suitable treatments would decrease the patients’ physical problems. Thavorcharoensap in Thailand and Ismail in Malaysia evaluated the QOL amongst patients aged under eighteen years using PedsQL 4.0 questionnaire and their results revealed that the QOL in those studies was better than ours in SF, EF and PF domains whereas Ismail came to similar results compared to our study, regarding PF domain.^1^,^12^ Such differences could originate from the differences in treatment methods, social culture,^3^ using different questionnaire or possibly difference in age structure of the patients.

The results have also demonstrated that sex is influential only in GH domain and the girls’ status seemed to be better. Although it sounds that problems resulting from thalassemia and its treatment are not any different between girls and boys, the expectations from the males in the society (such as managing a family, income, etc.) in Iran can justify these differences. In a study by Hadi, women had a better QOL in SF domain^11^ whereas studies conducted in Thailand and Malaysia have shown that sex is not effective on the QOL domains.^1^,^12^

There was no significant difference between the domains of QOL regarding age groups; however, in Thailand, it was presented that the teenage patients had a better QOL in comparison with younger patients.^1^ Such a point could originate from the difference in the age of the samples in the two studies; in Thailand, patients younger than eighteen years were evaluated and it goes without saying that compatibility with the conditions related to the disease has not been formed in younger ages and such a status is better accepted by the patient by pass of time.

Regarding the two types of thalassemia under study, the patients with thalassemia intermedia have worse QOL in PF and RLE domains; it may be due to the fact that thalassemia major patients who were too severe and had low quality of life might die since they were young. Those who were older than 15 and were recruited in this study were those who received appropriate care so they had higher quality of life. Meanwhile QOL in BP domain, was better in thalassemia intermedia; the patients with thalassemia intermedia were received fewer blood transfusion than the patients with thalassemia major and it may be reason that these patients had fewer bodily problems (i.e. pain) related to transfusion compared thalassemia major patients.

Another finding of this study was a better QOL found in those receiving oral chelators in MH and SF domains. Osborne et al., have also claimed that oral iron chelator would improve QOL amongst these patients.^13^

In this study, there was no correlation between Hb and ferritin level, before transfusion, and the different domains of QOL. In a study in Thailand, there was a correlation between the patients’ QOL and Hb level before transfusion and patients with a Hb level higher than 9mg/dl, before transfusion, had a better QOL whereas such a correlation was not found for ferritin level.^1^

Only a few patients reported to have complications and their QOL was not different from those without complications. Undoubtedly, this finding is difficult to interpret and could probably result from the small number of patients with complicationsa; hence, we need to increase the number of evaluations.

There are two limitations that need to be addressed regarding the present study. First, patients with thalassemia intermedia who referred to this center generally referred to receiving blood transfusion. This may the probable reason for high ferritin level in these patients. So the generalization of the findings should be made with caution. Second, regarding the high ferritin level in our patients it was expected that more than 5% of patients might had complications. The low percentage of complicate may be due to under reporting since in the study information about complications was collected based on self-report.

In general, the patients’ QOL was not acceptable, and the results imply that patients with thalassemia intermedia have worse status compared to those with thalassemia major. However, more large investigations are required to embody social issues of thalassemia patients.

## Figures and Tables

**Table 1:** Sociodemographic and clinical characteristics of the patients.

	N (%) / Mean (SD)
Gender	
Male	121 (39.3)
Female	187 (60.7)
Age (years)(n=306)	22.95 (4.82)
16–20	123 (40.2)
21–25	98 (32.0)
26–30	66 (21.6)
31–45	19 (6.2)
Educational Level (n=308)	
Illiterate	17 (5.6)
Below Diploma	117 (38.2)
Diploma	113 (36.9)
College	59 (19.3)
Income per month (n=305)	
Below 5milion Rials	267 (87.5)
5milion Rials & Above	38 (12.5)
Diagnosis	
Beta-Thalassemia major	209 (67.9)
Thalassemia intermedia	99 (32.1)
Hb level† (n= 282)	9.43(6.53–11.5)
Serum ferritin level‡ (n=262)	2673(212–9979.5)
Iron chelation treatment (n=301)	
Talassemia major	
Deferroxamine	183 (88.8)
Deferasirox	23 (11.2)
Thalassemia intermedia	
Deferroxamine	80 (84.2)
Deferasirox	15 (15.8)
Complication (n=304)	
Yes	15 (4.9)
No	289 (95.1)

† Pre-transfusion Hb level 4 measurement prior to QOL assessment [Median(Range)].

‡ Pre-transfusion Ferritin level 2 measurement prior to QOL assessment [Median(Range)].

**Table 2:** Quality of Life scores of patients and Healthy individuals in Iran.

SF-36 Domains	Total Patients (n=308)	Thalassemia Major (n=209)	Thalassemia Intermedia (n=99)	Healthy Individuals† (n=4804)
Physical Functioning*	67.95 (22.68)	72.11 (21.19)	59.19 (23.34)	85.2 (20.8)
Role Limitation-Physical	53.24 (18.58)	54.31 (17.88)	50.96 (19.89)	70.0 (38.0)
General Health	53.05 (16.96)	52.69 (17.42)	53.84 (15.99)	67.5 (20.4)
Vitality	55.02 (14.93)	54.31 (15.05)	56.57 (14.61)	65.8 (17.3)
Social Functioning	60.11 (20.89)	60.11 (20.84)	60.10 (21.10)	76.0 (24.4)
Role Limitation-Emotional*	55.13 (21.09)	57.89 (19.64)	49.32 (22.91)	65.6 (41.1)
Mental Health	56.91 (16.40)	56.22 (15.54)	58.40 (18.11)	67.0 (18.0)
Bodily Pain*	55.47 (20.96)	53.40 (20.48)	59.88 (21.38)	79.4 (25.1)

† Data from Montazeri A.^8^

* P< 0.05 between two groups (thalassemia major and intermedia).

**Table 3:** Quality of life scores of patients based on type of chelator.

SF-36 Domains	Injection (n=263)	Oral (n=38)	*P*-Value
Physical Functioning	68.59 (21.63)	63.55 (27.55)	NS
Role Limitation-Physical	52.62 (17.65)	56.74 (22.19)	NS
General Health	52.37 (16.56)	59.32 (15.26)	< 0.05
Vitality	54.33 (14.25)	59.87 (15.56)	< 0.05
Social Functioning	59.03 (20.29)	67.76 (22.06)	< 0.05
Role Limitation-Emotional	54.60 (19.94)	57.89 (26.63)	NS
Mental Health	56.30 (15.82)	61.84 (18.65)	0.05
Bodily Pain	54.30 (19.83)	62.79 (23.99)	< 0.05

**Table 4:** Result of the linear regression analysis (regression coefficient) relating study variables with the SF-36 Domains.

Variable	SF-36 Domains							

	PH	RLP	GH	V	SF	RLE	MH	BP
Age	−0.004	−0.082	−0.047	−0.086	−0.151	−0.048	−0.209	−0.229
Sex (Male^a^)	−4.569	−3.475	−6.593**	−4.277*	−3.484	−1.665	−1.215	0.096
Group (Major^a^)	−15.939**	−4.773	0.369	2.152	0.298	−13.333**	1.882	6.730*
Education (Below Diploma^a^)	−4.399	0.025	3.512	0.330	−7.062**	4.445	−1.094	−1.246
Income (Below 5million Rials^a^)	0.148	8.594*	2.247	4.538	6.460	−3.220	4.274	−0.765
Chelator (Injection^a^)	−3.922	2.990	5.728	5.355	10.803**	3.446	10.319**	10.131*
HB	1.742	1.708	0.009	0.672	2.653	−0.123	0.409	1.332
Ferretin	0.000	−0.001	0.000	0.000	0.000	0.000	0.000	0.000

A:

* *P*<0.005 ;

** *P*<0.01
